# The evolution of meiotic sex and its alternatives

**DOI:** 10.1098/rspb.2016.1221

**Published:** 2016-09-14

**Authors:** Ghader Mirzaghaderi, Elvira Hörandl

**Affiliations:** 1 Department of Agronomy and Plant Breeding, Faculty of Agriculture, University of Kurdistan, Sanandaj, Iran; 2 Department of Systematics, Biodiversity and Evolution of Plants, Georg-August-University of Göttingen, Göttingen, Germany

**Keywords:** paradox of sex, restitutional meiosis, apomixis, automixis, selfing

## Abstract

Meiosis is an ancestral, highly conserved process in eukaryotic life cycles, and for all eukaryotes the shared component of sexual reproduction. The benefits and functions of meiosis, however, are still under discussion, especially considering the costs of meiotic sex. To get a novel view on this old problem, we filter out the most conserved elements of meiosis itself by reviewing the various modifications and alterations of modes of reproduction. Our rationale is that the indispensable steps of meiosis for viability of offspring would be maintained by strong selection, while dispensable steps would be variable. We review evolutionary origin and processes in normal meiosis, restitutional meiosis, polyploidization and the alterations of meiosis in forms of uniparental reproduction (apomixis, apomictic parthenogenesis, automixis, selfing) with a focus on plants and animals. This overview suggests that homologue pairing, double-strand break formation and homologous recombinational repair at prophase I are the least dispensable elements, and they are more likely optimized for repair of oxidative DNA damage rather than for recombination. Segregation, ploidy reduction and also a biparental genome contribution can be skipped for many generations. The evidence supports the theory that the primary function of meiosis is DNA restoration rather than recombination.

## 1. Introduction

Meiosis is a key step in sexual reproduction and an ancestral, ubiquitous attribute of eukaryotic life cycles [1]. In the last decades, much progress has been made in understanding the mechanics of the different steps of meiosis [2], but still there is much discussion about the actual evolutionary advantage of meiotic recombination [3]. Meiosis is the major component of the evolutionary paradox that sex is maintained in eukaryotes despite the high costs of sexual reproduction [4–6]. The costs of meiosis include that recombination can break up favourable gene combinations, and that it is a time-consuming, risky process which is prone to errors [5]. The costs of biparental sexual reproduction include the need of two parental individuals for producing offspring, with all the efforts of mate searching, mate finding, risk exposure during mating, among others [5,6]. Strikingly, almost all forms of uniparental reproduction do maintain meiosis, but abandon just outcrossing. Hence, the paradox of sex in eukaryotes must focus on the purpose of meiosis.

Traditionally, genetic recombination as a consequence of meiosis was seen as a major evolutionary benefit of sex. However, empirical and theoretical research over the last century, strongly questioned this idea, and point at the high variability of possible cases under various selection scenarios [3]. Sex need not increase genetic variation in a population; genetic variation can be selected against and evolution need not favour increased levels of genetic exchange even if variability would be advantageous [3].

Other theories explain the primary function of meiosis for having a role in DNA restoration, either indirectly by elimination of deleterious mutations via natural selection [7], for directly repairing DNA double-strand breaks (DSBs) [8], or for removal of oxidative DNA damage in germline cells [9,10]. Prophase I would be needed for repair of DNA damage, while reductional division allows for elimination of mutations in the haploid phase [9,11].

**Figure 1.**
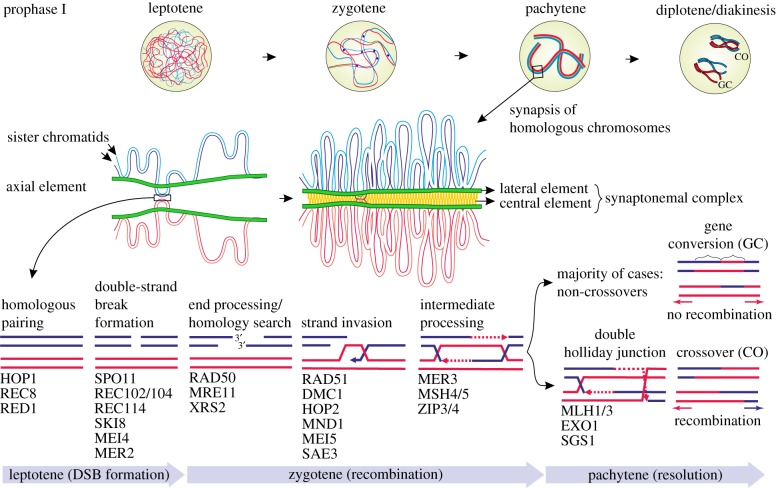
Processes during meiotic prophase I. Proteins in *Saccharomyces cerevisiae* (after [12–15]) involved in each phase are shown inside the figure. Most cases are resolved without recombination (exchange of flanking regions, see red versus blue arrows). (Online version in colour.)

In this review, we will present a novel view on this question by examining the steps of meiosis (figure 1) and how these are kept in naturally occurring modifications. Our rationale is that essential components and functions of meiotic sex should be conserved across eukaryotes and would occur in various variants of modes of reproduction, while the expression of less essential functions could be just facultative and context-dependent. We will review: (i) evolutionary origin and functions of the steps of meiosis; (ii) forms and genetic consequences of restitutional meiosis; (iii) current knowledge on apomixis and apomictic parthenogenesis; (iv) automixis and selfing; and finally (v) we will provide a synthesis of all aspects, presenting the novel view that the various modes of reproduction keep the functions as a DNA restoration tool, while mixis, as the main process creating recombination, can be more or less reduced or abandoned (table 1).

**Table 1.** Overview of modifications of main steps of meiosis and their evolutionary relevance in plants and animals. (Plant-specific proteins in italics [16], animal-specifics in bold face [17]. For yeasts, see figure 1.)

	homologue pairing	DSB formation, end processing, and strand invasion	crossover resolution	non-crossover resolution	segregation	meiosis II	gametogenesis and gametes	cross-fertilization of egg cell	variation in offspring	ploidy constancy in offspring
beneficial function	suppression of ectopic recombination	SPO11 antioxidant activity; chiasmata formation	DSB repair cohesion, recombination	DSB repair, cohesion, gene conversion	cohesion of homologues, suppression of S phase II	reductional division, variable meiotic products	unmasking deleterious mutations; purifying selection	restoration of diploidy, hetero-zygosity, allele diversity	individual genotypes hetero-zygosity	homologue pairing regular, gene copy no. constant
proteins in normal meiosis	HOP1 *MND1* *ASY1* REC8	**SPO11**, *SPO11-1/2/3*, **MEI1, MEI4**, MRE11, RAD50, NBS1 HOP2, MND1, DMC1, **REC114**, PRD1*,* PRD2, *PRD3, SKI8, SWI1, DFO*	MSH4/5, MER3, MLH1/3, *ZIP4*, MUS81, EME1 (MMS4)		*SGO1*, **SGO2**, *OSD1*, *TAM, SMG7, JAS, PS1*		*CDKA;1* *SWA1*			
normal meiosis	yes	yes	yes (minor proportion)	yes (major proportion)	yes	yes	yes	yes	yes	yes
FDR	yes	yes/no	yes/no	yes/no	yes	yes	no	yes	yes	no
SDR	yes	yes	yes	yes	yes	no	no	yes	yes	no
apospory	yes	yes	yes	yes	yes	yes / abortion somatic cell	yes /abortion	yes	recombinant clonal	yes
diplospory	yes	yes/no	yes/no	yes/no	yes	yes	no	no	clonal	yes
adventitious embryony	yes	yes	yes	yes	yes	yes	yes	yes somatic cell	recombinant clonal	yes yes
selfing	yes	yes	yes	yes	yes	yes	yes	no	loss of hetero-zygosity	yes
automixis	yes	yes	yes/no	yes/no	yes	yes	yes	no	loss of heterozygosity	yes
apomictic parthenogenesis	modified	modified	gene conversion	no	no	no	yes	no	clonality	yes

## 2. Origin of meiosis and DNA repair functions at prophase I

This section shows that processes at prophase I are most conserved in the evolution of eukaryotes, and that they probably evolved for DNA repair, but not for increasing recombination.

Meiotic sex already occurred in the last common ancestor of eukaryotes [18], and probably evolved out of bacterial transformation [19]. The primary evolutionary function of transformation may be the use of a homologous DNA molecule for recombinational repair of DNA DSBs and other physical damage caused by reactive oxygen species (ROS) [19,20]. Hence, an enzymatic DNA repair machinery already existed in prokaryotes which was taken over by eukaryotes [21]. DNA repair was badly needed in the first eukaryotes because of endogenous production of ROS with the onset of cellular oxygen respiration via (proto-) mitochondria [1]. Strong arguments for this hypothesis are that the core genes involved in meiosis have homologues in prokaryotes [22,23]. Several proteins belong to a ‘core’ meiosis-specific subset typically found in all eukaryotes [24] (figure 1).

Meiosis I could have originated for repair of DNA DSBs as a consequence of strong oxidative damage [8]. In many extant organisms, DSBs, introduced by the meiosis-specific spo11 protein, appear to be done regularly [25]. DSB formation is under control of numerous enzymes acting in complex feedback loops, and appears clustered in certain hotspots [12,26]. However, only a minimum of DSBs is required for correct chromosomal segregation at anaphase I [27]. Strikingly, recent meiosis research across all eukaryotes observed that DSB formation outnumbers by far crossover formation, with the remaining events repaired as non-crossovers or via intersister repair (e.g. [16]). Non-crossovers do not result in recombination (exchange of flanking regions), but often give rise to gene conversion (figure 1). Recombination tends to occur in regions of the chromosomes where the DNA is only loosely packaged, not heavily methylated, and also near the start of genes [28]. Hence, programmed DSB formation might have not evolved ‘for a purpose’ of recombination, but for scavenging previously existing DNA radicals by the tyrosine-end of spo11 [10]. In support of this hypothesis, facultative asexual eukaryotes increase frequencies of sex under ROS-generating stress conditions (electronic supplementary material, S1). Abiotic stress triggers sex in plants [10,29–31], and DNA damaging agents increased meiotic recombination in yeast, nematodes and fruit flies [19].

Meiotic repair of oxidative lesions is restricted to germline cells, probably because of the risks of failure of DSB formation [26], the costs of producing proteins (figure 1) and also abundant ATP [19]. These risks and costs are especially high for protists, but can be lowered for multicellular organisms which can differentiate in germline and somatic cells ([5,11]; electronic supplementary material, S2). The immediate selective advantage for multicellular eukaryotes is that only immortal germline cells undergo an intense removal of DNA radical damage without involving other, less expensive, but potentially mutagenic non-homologous repair mechanisms which suffice for mortal somatic cells (e.g. [32]). Hence, meiotic repair directly increases DNA quality of offspring. Mutants in key meiosis proteins remain sterile [19,32] and would be in nature eliminated by truncating selection. Because of the reciprocal nature of meiosis, the benefit of DNA repair will apply to *all* offspring of *both* parental individuals [33]. Under these auspices, selection will strongly favour homologous recombinational repair irrespective of amounts of recombination arising from the process. In various modes of reproduction, homologue pairing, DSB formation and subsequent DNA break repair (figure 1) is the least dispensable step in eukaryotic modes of reproduction (table 1).

## 3. Elimination of deleterious mutations via ploidy reduction

**Figure 2.**
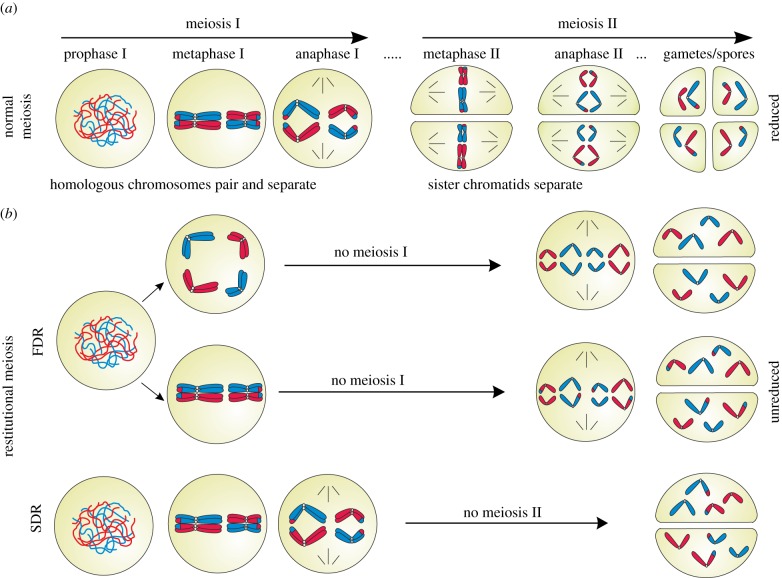
Normal (*a*) and non-reductional (*b*) meiotic divisions resulting in reduced (*n*) and unreduced (2*n*) meiotic products (gametes/spores) for a diploid parent with two chromosome pairs. Maternal and paternal chromosomes are shown in red and blue, respectively. FDR and SDR maintain different levels of parental heterozygosity. (Online version in colour.)

During meiosis a single round of DNA replication occurs followed by two successive rounds of chromosome segregation, resulting in haploid meiotic products (figure 2*a*). Ploidy reduction provides an efficient mechanism to expose deleterious mutations to purifying selection [9,11]. In a diploid stage, deleterious recessive mutations can be ‘masked’, i.e. they would not be expressed because a functional gene copy is available at the homologous chromosome [34,35]. Consequently, such mutations would not be exposed to natural selection and thus would accumulate in the long term. Ploidy reduction will lead to expression of the mutated genes and expose the gametes carrying them to purifying selection, and selection is most efficient in haploids [36]. Gametes carrying deleterious mutations, even if viable, are unsuccessful in the fertilization process because of the competition with non-mutated gametes [11,37].

Mutation accumulation is a long-term process, and effects of mutations depend also on epistatic interactions (e.g. [38]). In an asexual lineage, deleterious mutations would accumulate in a ratchet-like manner because without recombination, the least loaded class of offspring cannot be restored (Muller's ratchet; [7]). Hence, the ploidy reduction would be expected to be under a more relaxed selective pressure in the short term, and should not be essential for each and every generation.

## 4. Meiotic restitution, unreduced gametes and polyploidy

In fact, ploidy reduction in gametes is a disposable, non-conservative step. Unreduced gamete formation largely represents a broad array of quite flexible, environment-dependent modifications of meiosis, with different underlying mechanisms (figure 2*b*). Homologue pairing, DSB formation and DNA repair are kept in most cases, but just the ploidy reduction and the elimination of mutations are abandoned. Unreduced gametes can transfer such ‘masked’ mutations to the polyploid offspring, where mutation is even better buffered. Hence, restitution has no short-term negative effects, but rather facilitates accumulation of mutations over many generations [3]. Although regular homologue pairing is difficult for newly formed polyploids, the process of diploidization in plants demonstrates that selection favours re-ordering of homologue pairing rather than skipping the prophase of meiosis I.

Restitutional meiosis is a mechanism which results in unreduced (2*n*) gametes, either by the first division restitution (FDR, skipping meiosis I), or by second division restitution (SDR, skipping meiosis II). In FDR-type mechanisms, the meiotic cell division is completely converted into a mitotic division generating 2*n* gametes with full parental heterozygosity (figure 2*b*). However, in some types of FDR, meiosis I is not completely omitted and the resulting 2*n* gametes transmit 70–80% of the parental heterozygosity [39]. In SDR mechanisms, however, meiosis I with its repair functions proceeds normally; consequently, the resulting 2*n* gametes retain around 30–40% of parental heterozygosity [39] at the telomeric side of crossing over (figure 2*b*). In interspecific hybrids, a reductional division of bivalents together with an equational segregation of univalents can give rise to unreduced gametes (indeterminate type of meiotic restitution [40]).

Possible cytological mechanisms resulting in FDR or SDR pathways include defects in meiotic cell plate formation and cytokinesis, complete omission of the first or the second meiotic division, or defects in spindle formation or function [41–43]. Moreover, mutations in the regulators of the key transitions during meiosis (prophase to meiosis I, and meiosis I to meiosis II) can result in unreduced gamete formation [44]. Unreduced gamete formation in natural populations usually is a consequence of temperature shocks [45–47]. Extreme temperatures can disturb gene expression and the enzymatic machinery during meiosis at many different steps, whereby cold and heat have different underlying mechanisms (electronic supplementary material, S1). Unreduced gametes can also be produced by pre-meiotic or post-meiotic genome doubling ([48] electronic supplementary material, S1), whereby the DNA repair aspect of meiosis is retained.

The consequence of unreduced gametes formation is polyploidy [47,49,50] (electronic supplementary material, S3). While polyploidy is very common among plants, it is in vertebrates only observed among fishes and frogs [51]. Strikingly, meiosis in polyploid plants maintains homologue pairing, DSB formation and repair via different mechanisms, despite the difficulties of a regular pairing and segregation of a higher number of chromosomes [52]. Since selection for fertility usually increases frequencies of bivalent formation over generations, polyploid lineages gradually convert to diploids with regular cytological behaviour accompanied by genetic differentiation of duplicated loci (‘diploidization’, [52,53]; electronic supplementary material, S3). Backcrossing or selection for transgressive segregants might increase fertility [54]. In the long term, polyploidization is not at all a pathway doomed to extinction. All angiosperm species have had at least one historical polyploidy event [55,56]. Whole-genome duplication has been recognized as an important factor for diversification of eukaryotes [57].

## 5. Apomixis: a little bit of sex

Most forms of asexual reproduction do keep meiosis either in a facultative sexual pathway or in an altered form, maintaining both repair functions and mutation elimination to some degree. Protists usually alter between mitotic (asexual) and meiotic (sexual) reproduction (electronic supplementary material, S2), while multicellular eukaryotes show a variety of asexual developmental pathways.

**Figure 3.**
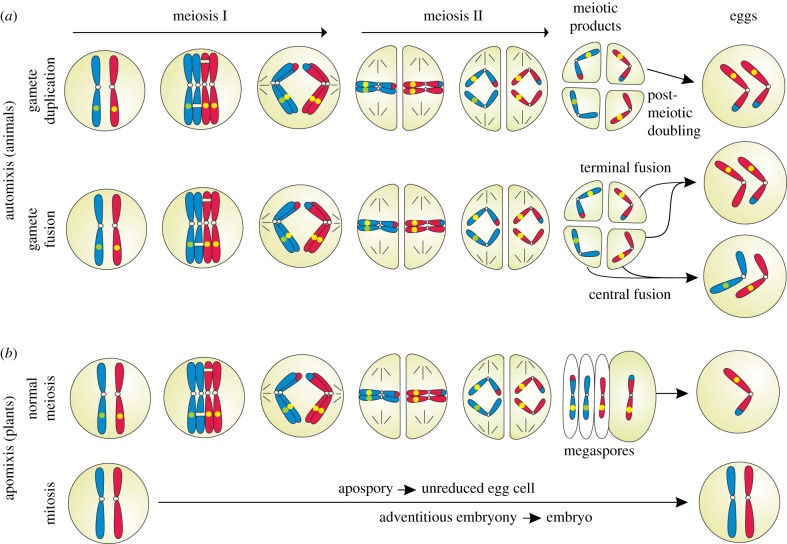
Forms of asexual reproduction with meiosis; unreduced eggs develop parthenogenetically. (*a*) Automixis in animals keeps female meiosis, but restores diploidy either via gamete duplication or via fusion of meiotic products, followed by loss of heterozygosity. (*b*) Apomixis in plants has three major pathways: in apospory and adventitious embryony, the meiotic pathway runs in parallel to somatic development, whereby reduced egg cells are being fertilized. Diplospory involves a female restitutional meiosis either via FDR or SDR (figure 2). Gene conversion is indicated by green and yellow circles on homologous chromosomes. (Online version in colour.)

In angiosperms, apomixis (reproduction via asexually formed seeds [58]) is found naturally in *ca* 2.2% of genera [59] and represents various modifications of female sexual development [60] (electronic supplementary material, S4). Male meiotic development is usually not altered [61], and functional pollen is needed for *ca* 90% of species for fertilization of polar nuclei and proper endosperm formation [62]. Strikingly, natural apomictic plants hardly ever lack meiotic sex completely. In adventitious embryony, both sexual and apomictic seedlings are formed within the same seed (polyembryony [63]). In facultative gametophytic apomicts, varying proportions of sexual seed are formed in parallel to apomictic ones [29,61,64–66] (figure 3; electronic supplementary material, S3). In the former, repair functions and purifying selection against deleterious mutations can act efficiently in the meiotically reduced gametophytes [37]. This mechanism probably counteracts mutation accumulation in facultative apomicts [67], whereas obligate asexual systems like permanent translocation heterozygosity do show the expected mutation accumulation [68].

Apomixis represents a genetic and epigenetic deregulation of the sexual pathway [60,69–71] and arises from the action of a few, usually dominant alleles or epialleles [72]. Apomixis has been induced by mutation in genes with different functions, including epigenetic regulation through small non-coding RNA pathways [73], DNA methylation [74] or encoding RNA-helicase [75]. Such a deregulation of sexual pathways has been hypothesized to be a consequence of hybridization and/or polyploidization [60,76,77].

In contrast to angiosperms, the vast majority of apomictic ferns (*ca* 10% of species) are reported to be obligate asexual [78] owing to non-functional archegonia [62,79]. The major reproductive pathway is via pre-meiotic doubling, followed by a normal meiosis producing diploid spores; the resulting gametophytes produce a new fern from a somatic cell without fertilization [62]. Hence, both recombinational repair and ploidy reduction takes place, only fertilization is abandoned which is problematic for ferns because of the dependence on water. Some fern species have an apomictic-like development as shown in the electronic supplementary material, S4 [62]. Despite obligate apomixis, there is no evident selective disadvantage as speciation/extinction rates of sexual and asexual ferns do not differ [79].

Apomictic parthenogenesis in animals involves suppression of meiosis, and mitosis-like cell divisions resulting in genetically maternal offspring. This form of reproduction is also mostly facultative (*tychoparthenogenesis*) and mainly found in invertebrates (rotifers, many arthropods; [80]). As in plants, clonal turnover may counteract the loss of clonal fitness over time [81]. Obligate apomixis is rare and occurs, e.g. in bdelloid rotifers, in which neither meiosis nor males have occurred for millions of years [82]. Here, meiosis is replaced by gene conversion among four collinear chromosome sets [83]. Gene conversion either replaces a segment carrying a mutated allele with an unmutated copy, or makes the mutated allele homozygous and hence exposes it to purifying selection. Gene conversion limited significantly accumulation of deleterious mutations and allelic sequence divergence (Meselson effect, see [84]). Further, enrichment of genes involved in resistance to oxidation, carbohydrate metabolism and regulation of transposable elements was observed, probably to cope with environmentally induced oxidative stress [83]. Hence, meiosis is only dispensable if alternative DNA restoration mechanisms are available.

## 6. Meiosis, but no mates: automixis, selfing and intragametophytic selfing

Many forms of uniparental reproduction do keep meiosis, but just abandon outcrossing. The genetic variation arising from fusion of genetically different gametes appears to be dispensible for offspring production. Importantly, Mendelian assorting and gametic recombination (by segregation and later on fusion of genetically different gametes) contribute quantitatively much more to genetic variation than meiotic recombination produced by crossovers [34]. Strikingly, this variation-creating process is much more often skipped than meiosis itself. An important question regards the selective value of complementation or the heterosis contribution from two parents.

Most parthenogenetic animals reproduce via automixis [80]. Diploid gamete formation is achieved either by fusion of products of the same meiosis, or by post-meiotic doubling of chromosome sets (figure 3). The unreduced oocyte develops parthenogenetically, which means that a single female can produce offspring (for details, see [85]). Parthenogenesis may also remain facultative, emerging just occasionally in isolated females, as it was observed in reptiles [86,87], in insects [88] and in fishes [89]. Meiosis I is kept in all three major forms of automixis (figure 3), but often results in increased homozygosity. Complete homozygosity arises in the offspring of automictic animals at centromeric regions, independently of mode of automixis, while in centromere-distant regions recombination can take place [90]. The rapid loss of heterozygosity leads to inbreeding depression because of expression of previously masked, deleterious recessive alleles. This loss of complementation has greater disadvantages than costs of meiosis [91].

Automixis is further constrained by certain sex determination systems, when automictic females can produce just male offspring [92]. In water-fleas (*Daphnia*), parthenogenesis is automictic with predominant terminal fusion [90]. Obligate parthenogenesis starts with meiotic homologue pairing, but without homologous recombination, and is continued with a mitotic-like division [93]. In cyclical parthenogenesis, parthenogenetic egg formation is followed by a stress-induced sexual cycle, where meiotically produced resting eggs are being fertilized by haploid males. A genomic inventory of *Daphnia* revealed that all meiosis genes are present in parthenogenetic species, but often in multiple copies. Expression patterns of most genes were similar in meiosis and parthenogenesis, but differed just in expression levels [93]. Numerous paralogues showed divergent expression patterns under different environmental conditions [94].

Strikingly, meiosis is kept in automictic animals, despite the fact that automixis can result in loss of heterozygosity and inbreeding depression, or in male offspring only. Selection for keeping repair functions at meiosis I is obviously stronger than selection for heterozygosity. Just the mechanism of mutation elimination during the short haploid phase might be weakened. Automixis can be even lost again, as reversals from automictic asexuality to obligate sexuality occurred in Oribatid mites [95].

Selfing in angiosperms involves independent male and female meioses, and formation and fusion of both male- and female-reduced gametes on the same individual. Cytologically, selfing is more similar to automixis in animals as in both cases the same parental chromosome set is reshuffled; continued selfing results in loss of heterozygosity by 50% per generation. Selfing is in angiosperms repeatedly gained [96] and performed facultatively by *ca* 40% of species. Successful selfing requires only that flower morphology and timing of development allows self-pollination, and breakdown of self-incompatibility (SI) systems which would inhibit pollen tube growth. SI systems have genetic control mechanisms acting independently from meiosis [97]. Homosporous ferns can self-fertilize on bisexual gametophytes [98], which produces completely homozygous sporophytes in a single generation. However, polyploid gametophytes can reduce inbreeding depression [99], which explains the preference of polyploid homosporous ferns for gametophytic selfing [100]. Intragametophytic selfing occurs also in bryophytes, but little is known about frequencies and evolutionary implications [62].

Uniparental reproduction is favoured in the short term owing to gene transmission advantages, improved colonization ability [101] and reproductive assurance under rare mate conditions [102,103]. The main factor disfavouring a transition to permanent selfing is loss of heterozygosity and inbreeding depression [104], causing reduced diversification and long-term risk of extinction [105].

## 7. Synthesis and outlook

Meiosis is an ancient and indispensable feature of eukaryotic life. Almost all forms of asexual and uniparental reproduction in eukaryotes represent just modifications of meiosis (table 1). Complete and long-term silencing of meiosis, as in ancient asexual bdelloids, is extremely rare and requires alternative mechanisms to cope with environmentally induced oxidative stress, and with elimination of deleterious mutations. Ploidy reduction can be avoided in the short term, resulting in polyploidization. Interestingly, selection favours in sexual polyploids a process of returning to a regular pairing of chromosomes at meiosis I rather than skipping the process. Many forms of uniparental reproduction do exist with meiosis, but without biparental sex. Loss of genetic variation by loss of outcrossing appears to be much less critical for further development and evolution than a complete absence of meiosis. There is obviously no immediate selective pressure to maintain outcrossing, although in the long term the loss of heterozygosity and its negative effects must be somehow compensated.

Hence, we propose the view that the key step of prophase I, i.e. homologue chromosome pairing, DSB formation and DNA strand exchange, even without crossing over formation and recombination, is the main indispensable, ancestral and highly conserved process in eukaryotic life cycles. This step must be maintained by a very strong selective pressure, as failure at this phase usually results in sterility or reduced fertility. But, this process cannot be maintained by selection on variable offspring only, as it results in few actual recombination events (crossovers), while many more initial DSBs are formed. Many arguments support the theory that the primary function of meiosis is DNA restoration rather than recombination [11]: first, meiosis is not at all optimized to create new allele combinations; second, meiosis is responsive to environmental stress which causes oxidative stress in tissues in various ways; third, repair of oxidative damage is an indispensable ‘must’ for cellular survival, while recombination is not; fourth, reduction of ploidy in gametes is the most efficient way to purge deleterious mutations; however, this step can be skipped in the short term; fifth, mixis can be easily abandoned; finally, many successful forms of ‘a little bit of sex’, i.e. facultative apomixis, and facultative or cyclical parthenogenesis exist, with a reduction of recombination and of genetic diversity in the offspring.

Under these auspices maintenance of sex is no longer a paradox because meiosis appears to be indispensable for eukaryotic reproduction. It is no surprise that shifting from established sex to asexuality is constrained in many different aspects (multigenic control, group-specific developmental pathways [11,92,106]). Future research on the topic should be interdisciplinary and focus on detailed cytological and developmental studies, accompanied by transcriptomic and genomic studies. Genomics has opened new avenues for collecting empirical data on mutation accumulation and their effects. Experimental and biochemical work is needed to understand the stress-sensitivity of meiosis and the connection to compensation of oxidative stress and maintenance of cellular redox homeostasis. Mathematical modelling on recombination and mutation needs to take into account the complexity of meiosis and its multigenic control, the different cytological steps of meiosis, and the many different forms of asexual reproduction which maintain some but not all aspects of meiosis–mixis cycles.

## Supplementary Material

Supplement 1. Examples of meiotic restitution and changes resulting from environmental stimuli in various species.

## Supplementary Material

Supplement 2. Scheme of unicellular and multicellular eukaryotic life cycles

## Supplementary Material

Supplement 3: Polyploidization.

## Supplementary Material

Supplement 4: Mechanisms of sexual and apomictic reproduction in flowering plants.

## Data accessibility

Data available at https://dx.doi.org/10.6084/m9.figshare.c.3457461.
