# Supplementary material for: The evolution of meiotic sex and its alternatives
Source: Proc Biol Sci. 2016 Sep 14;283(1838):20161221. doi: 10.1098/rspb.2016.1221 (PMC5031655; doi:10.1098/rspb.2016.1221)
Supplement: Supplement 1. Examples of meiotic restitution and changes resulting from environmental stimuli in various species. [file rspb20161221supp1.pdf]

Supplement 1. Examples of meiotic restitution and changes resulting from environmental stimuli in various species. FDR = first division restitution; SDR = second division restitution**.**

Type of restitution or effect	Cytological mechanism (A)	Studied organ	Taxon	Stimulus (B)	Reference (See Supplement 1)
FDR or SDR	1 or 2	female	*Boechera holboelli* (rockcress)	8	[1]
FDR, SDR	1, 2, 9	male	Orchidaceae (Orchids)	8	[2]
FDR	1, 5	male	*Triticum-Aegilops* (wheat) hybrid	4	[3]
FDR	4	male	*Triticum* (wheat thermo sensitive genic male sterile line)	2	[4, 5]
FDR	6	female	*Populus adenopoda* (poplar)	5	[6]
Asynapsis	6	male	*Caenorhabditis elegans* (nematode)	5	[7]
Asynapsis or abnormal synapsis	6	male	*Allium ursinum* (broad-leaved garlic)	5	[8]
Desynapsis	6	male	*Solanum melongena, S. violaceum, S. torvum* (aubergine and congeners)	9	[9]
SDR	3	male	*Dianthus caryophyllus* (carnation)	9	[10]
SDR	3	male	*Arabidopsis thaliana* (thale cress)	2	[11]
SDR	3	male	*Rosa sp.* (Rose)	5	[12]
SDR	3	male	*Arabidopsis thaliana* (thale cress)	6	[13]
SDR	ND	female	*Citrus* (lemons) interspecific hybrids	8	[14, 15]
Pre-meiotic doubling	7	male	*Nicotiana tabaccum* (Tobacco)	8	[16]
Pre-meiotic doubling	ND	male	*Turnera* interspecific hybrid	8	[17]
Pre-meiotic doubling	ND	male	*Chrysanthemum* (Chrysantheme)	8	[18]
meiosis II nondisjunction	8	male	Tipulidae (Crane fly)	2	[19]

Intermediate meiotic restitution	9	male	*Lilium* (Lily) hybrids	4	[20]
Post-meiotic gamete doubling	10	female	*Populus* (poplar)	1	[21]
Post-meiotic doubling gamete and SDR	10	female	diploid *Solanum* interspecific (potato) clones	8	[22]
Change in chiasma frequency	11	male	(*Schistocerca gregaria*) grasshopper	3	[23]
Dyad formation	ND	male	*Dasypyrum villosum* (mosquitograss)	2	[24]
Increased pollen size	ND	male	*Brassica* hybrids (mustard hybrids)	2	[25]
Increased pollen size	ND	male	*Rosa* (Rose) interspecific hybrids	5	[26]
Increased pollen size	ND	male	*Diospyros kaki* (Kaki tree)	2	[27]
Proportion of meiotic ovules increased	ND	female	*Paspalum* (Bahiagrass relative)	7	[28]
Proportion of meiotic ovules increased	ND	female	*Ranunculus auricomus* (goldilocks)	7	[29]
Proportion of sexual colonies increased	ND	female	*Volvox carteri* (green alga)	5	[30]
Varied degree of sterility in different populations	ND	male	*Drosophila melanogaster* (common fruit fly)	2 and 5	[31]

Cytological mechanism: **A: 1**) complete omission of first meiotic division, **2**) complete omission of second meiotic division, **3**) Impared spindle orientation in MII, 4) defects in meiotic cell plate formation in MI and cytokinesis, **5**) defect in meiotic cell plate formation in MII, **6)** Assembly prevention or disruption of synaptonemal complex, **7**) Cytomixis (migration of the nuclei from one plant cell to another through intercellular channels), **8**) chromosome malorientation, **9)** mix of univalents and bivalents that divide equationally and reductionally, ND: not determined; Stimulus **B: 1)** chemically induced, **2)** cold, **3)** environmental variation, **4)** haploidy or wide hybridity dependent, **5)** heat, **6)** mutation, **7)** prolonged photoperiod, **8)** spontaneous, **9)** varied in different seasons.

Supplement 1: References cited.

[1] Bocher, T.W. 1951 Cytological and embryological studies in the amphi-apomictic *Arabis holboellii* complex. *Kong. Danske Vidensk. Selsk. Biol. Skr.* **6**, 1–59.

[2] Teoh, S. 1984 Polyploid spore formation in diploid orchid species. *Genetica* **63**, 53-59.

[3] Fakhri, Z., Mirzaghaderi, G., Ahmadian, S. & Mason, A.S. 2016 Unreduced gamete formation in wheat × *Aegilops* spp. hybrids is genotype specific and prevented by shared homologous subgenomes. *Plant Cell Reports*, **35**, 1143-1154.

[4] Tang, Z., Zhang, L., Yang, D., Zhao, C. & Zheng, Y. 2011 Cold stress contributes to aberrant cytokinesis during male meiosis I in a wheat thermosensitive genic male sterile line. *Plant, Cell and Environment* **34**, 389-405.

[5] Xu, C., Liu, Z., Zhang, L., Zhao, C., Yuan, S. & Zhang, F. 2013 Organization of actin cytoskeleton during meiosis I in a wheat thermo-sensitive genic male sterile line. *Protoplasma* **250**, 415-422.

[6] Lu, M., Zhang, P. & Kang, X. 2013 Induction of 2n female gametes in *Populus adenopoda* Maxim by high temperature exposure during female gametophyte development. *Breeding Science* **63**, 96-103. (doi:10.1270/jsbbs.63.96).

[7] Bilgir, C., Dombecki, C.R., Chen, P.F., Villeneuve, A.M. & Nabeshima, K. 2013 Assembly of the synaptonemal complex Is a highly temperature-sensitive process that is supported by PGL-1 during *Caenorhabditis elegans* meiosis. *G3: Genes|Genomes|Genetics* **3**, 585-595. (doi:10.1534/g3.112.005165).

[8] Loidl, J. 1989 Effects of elevated temperature on meiotic chromosome synapsis in *Allium ursinum*. *Chromosoma* **97**, 449-458.

[9] Karihaloo, J. 1991 Desynapsis due to temperature stress in three species of *Solanum* L. *Cytologia* **56**, 603-611.

[10] Zhou, X., Mo, X., Gui, M., Wu, X., Jiang, Y., Ma, L., Shi, Z., Luo, Y. & Tang, W. 2015 Cytological, molecular mechanisms and temperature stress regulating production of diploid male gametes in *Dianthus caryophyllus* L. *Plant Physiology and Biochemistry* **97**, 255-263. (doi:http://dx.doi.org/10.1016/j.plaphy.2015.10.003).

[11] De Storme, N., Copenhaver, G.P. & Geelen, D. 2012 Production of diploid male gametes in *Arabidopsis* by cold-induced destabilization of postmeiotic radial microtubule arrays. *Plant physiology* **160**, 1808-1826.

[12] Pécrix, Y., Rallo, G., Folzer, H., Cigna, M., Gudin, S. & Le Bris, M. 2011 Polyploidization mechanisms: temperature environment can induce diploid gamete formation in *Rosa* sp. *Journal of Experimental Botany* **62**, 3587-3597.

[13] d'Erfurth, I., Jolivet, S., Froger, N., Catrice, O., Novatchkova, M., Simon, M., Jenczewski, E. & Mercier, R. 2008 Mutations in AtPS1 (*Arabidopsis thaliana* parallel spindle 1) lead to the production of diploid pollen grains. *PLoS Genetics* **4**, e1000274.

[14] Cuenca, J., Aleza, P., Juárez, J., García-Lor, A., Froelicher, Y., Navarro, L. & Ollitrault, P. 2015 Maximum-likelihood method identifies meiotic restitution mechanism from heterozygosity transmission of centromeric loci: application in citrus. *Scientific Reports* **5**, 9897. (doi:10.1038/srep09897).

[15] Esen, A., Soost, R.K. & Geraci, G. 1979 Genetic evidence for the origin of diploid megagametophytes in *Citrus*. *Journal of Heredity* **70**, 5-8.

[16] Mursalimov, S.R. & Deineko, E.V. 2015 How cytomixis can form unreduced gametes in tobacco. *Plant Systematics and Evolution* **301**, 1293-1297.

[17] Fernández, A. & Neffa, V.G.S. 2004 Genomic relationships between *Turnera krapovickasii* (2x, 4x) and *T. ulmifolia* (6x)(Turneraceae, Turnera). *Caryologia* **57**, 45-51.

[18] Kim, J.S., Oginuma, K. & Tobe, H. 2009 Syncyte formation in the microsporangium of *Chrysanthemum* (Asteraceae): a pathway to infraspecific polyploidy. *Journal of Plant Research* **122**, 439-444.

[19] Janicke, M.A., Lasko, L., Oldenbourg, R. & LaFountain, J.R. 2007 Chromosome malorientations aftermeiosis II arrest cause nondisjunction. *Molecular Biology of the Cell* **18**, 1645-1656. (doi:10.1091/mbc.E06-10-0963).

[20] Lim, K.B., Ramanna, M.S., de Jong, J.H., Jacobsen, E. & van Tuyl, J.M. 2001 Indeterminate meiotic restitution (IMR): a novel type of meiotic nuclear restitution mechanism detected in interspecific lily hybrids by GISH. *Theoretical and Applied Genetics* **103**, 219-230.

[21] Wang, J., Kang, X.Y., Li, D.L., Chen, H.W. & Zhang, P.D. 2010 Induction of diploid eggs with colchicine during embryo sac development in Populus. *Silvae Genetica* **59**, 40-48.

[22] Bastiaanssen, H.J.M., van den Berg, P.M.M.M., Lindhout, P., Jacobsen, E. & Ramanna, M.S. 1998 Postmeiotic restitution in 2n egg formation of diploid potato. *Heredity* **81**, 20-27.

[23] Shaw, D. 1971 Genetic and environmental components of chiasma control. *Chromosoma* **34**, 281-301.

[24] Stefani, A. & Colonna, N. 1996 The influence of temperature on meiosis and microspores development in *Dasypyrum villosum* (L.) P. Candargy. *Cytologia* **61**, 277-283.

[25] Mason, A.S., Nelson, M.N., Yan, G. & Cowling, W.A. 2011 Production of viable male unreduced gametes in *Brassica* interspecific hybrids is genotype specific and stimulated by cold temperatures. *BMC plant biology* **11**, 103.

[26] Crespel, L., Le Bras, C., Relion, D., Roman, H. & Morel, P. 2015 Effect of high temperature on the production of 2n pollen grains in diploid roses and obtaining tetraploids via unilateral polyploidization. *Plant Breeding* **134**, 356-364. (doi:10.1111/pbr.12271).

[27] Yamada, A., Tao, R. & Sugiura, A. 2005 Influence of low temperature before flowering on the occurrence of unreduced pollen in Japanese persimmon (*Diospyros kaki* Thunb.). *HortScience* **40**, 24-28.

[28] Quarin, C.L. 1986 Seasonal changes in the incidence of apomixis of diploid, triploid, and tetraploid plants of *Paspalum cromyorrhizon*. *Euphytica* **35**, 515-522.

[29] Klatt, S., Hadacek, F., Hodač, L., Brinkmann, G., Eilerts, M., Hojsgaard, D. & Hörandl, E. 2016 Photoperiod extension enhances sexual megaspore formation and triggers metabolic reprogramming in facultative apomictic *Ranunculus auricomus*. *Frontiers in Plant Science* **7**. (doi:10.3389/fpls.2016.00278).

[30] Nedelcu, A.M. & Michod, R.E. 2003 Sex as a response to oxidative stress: the effect of antioxidants on sexual induction in a facultatively sexual lineage. *Proceedings of the Royal Society of London B: Biological Sciences* **270**, S136-S139.

[31] David, J., Araripe, L., Chakir, M., Legout, H., Lemos, B., Petavy, G., Rohmer, C., Joly, D. & Moreteau, B. 2005 Male sterility at extreme temperatures: a significant but neglected phenomenon for understanding *Drosophila* climatic adaptations. *Journal of evolutionary biology* **18**, 838-846.
