# Supplementary material for: The evolution of meiotic sex and its alternatives
Source: Proc Biol Sci. 2016 Sep 14;283(1838):20161221. doi: 10.1098/rspb.2016.1221 (PMC5031655; doi:10.1098/rspb.2016.1221)
Supplement: Supplement 2. Scheme of unicellular and multicellular eukaryotic life cycles [file rspb20161221supp2.pdf]

Supplement 2. Scheme of unicellular and multicellular eukaryotic life cycles (left, e.g. *Saccharomyces cerevisiae*; right, e.g. an diplontic animal with early germline separation). Red, meiotic products; blue, cells dividing by mitosis; darker blue symbolizes accumulated DNA damage (oxidative lesions and mutations). Costs of sex differ between the two major types of eukaryotes (+, advantage, - disadvantage). In protists no division of labour is possible, but all cells are capable of asexual reproduction (in yeast, via budding) via mitosis. Meiosis probably represents just a risky repair tool in situations of high stress and starvation. In multicellular organisms, germline and somatic cells can act differentially. Somatic cells use mitosis to optimize growth and cell differentiation. The costly meiotic repair is restricted to the potentially immortal germline cells during prophase I, guaranteeing a high quality DNA for gametes and the new zygote which is essential for a more complex organism. This infers that a second parent is needed for reproduction. See a more detailed discussion in Hörandl (2009) [11]. Yeast life cycle after Herskovitz 1988 Life cycle of the budding yeast *Saccharomyces cerevisiae*. Microbiol. reviews 52, 536-553.

unicellular   multicellular

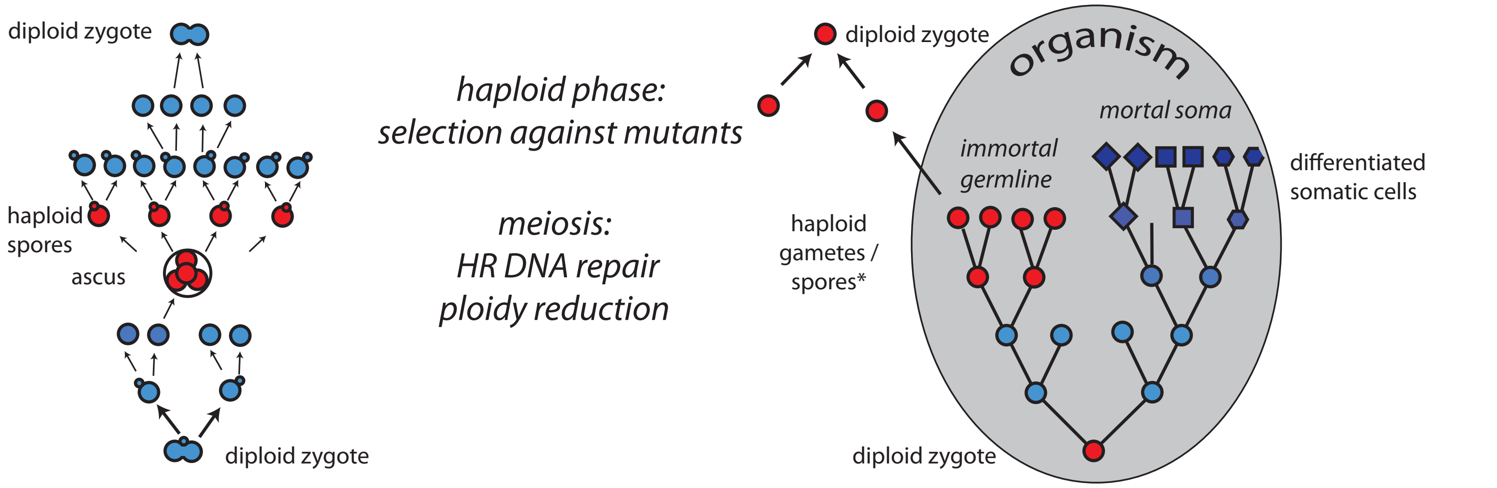

- meiosis (repair) and mitosis (growth) alternating in sexual/asexual generations	+ meiosis (repair) and mitosis (growth) can run in parallel in the same organism
- no cell differentiation; transcription and DNA synthesis inactive during meiosis	+ cell differentiation of somatic cells
- high (lethal) risk of meiosis failure	+ many meioses possible
- direct environmental influence on cells	+ germline better protected by other tissues
- homologous recombinational (HR) repair long before mating	+ homologous recombinational (HR) repair directly before mating; DNA of zygote was repaired and purged from mutants
+ all cell types can reproduce via mitosis	- only germline cells can reproduce; somatic cells are mortal
+ no cost of males	- cost of males (animals) or male functions (plants)
+ high quantity of offspring, selection eliminates damaged / mutated ones	- low quantity of offspring, but high DNA quality in zygote needed
predominant asexuality	predominant sexuality

*land plants produce spores after meiosis, which develop into haploid gametophytes; this generation produces then gametes
