# Supplementary material for: The evolution of meiotic sex and its alternatives
Source: Proc Biol Sci. 2016 Sep 14;283(1838):20161221. doi: 10.1098/rspb.2016.1221 (PMC5031655; doi:10.1098/rspb.2016.1221)
Supplement: Supplement 3: Polyploidization. [file rspb20161221supp3.pdf]

Supplement 3: Polyploidization. Natural polyploids are suddenly formed via unreduced gametes through four different pathways of homoploid bridge, unilateral pathway (triploid bridge), bilateral pathway and somatic chromosome doubling. Whether the result is an auto or allopolyploid depends on the level of homology between parental chromosomes. Allopolyploids as depicted here usually show a more regular homolog pairing than autopolyploids, as doubled chromosomes can pair. Hence, processes at meiosis are maintained in sexual polyploids despite higher chromosome numbers. Intense genome reorganization and return to bivalent formation in the newly formed polyploids (neopolyploids), a process known as diploidization, gradually leads to emergence of paleopolyploids.

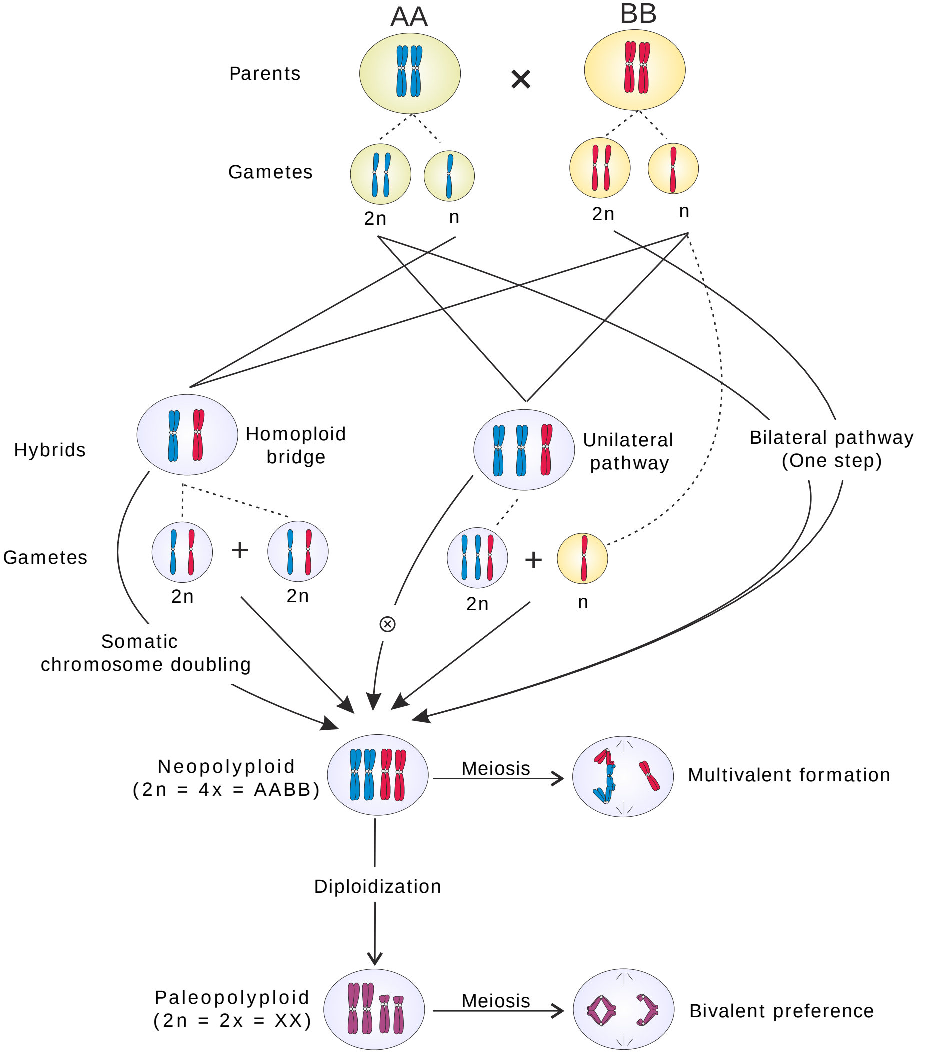

AA
BB
Parents
×
Gametes
2n
n
2n
n
Hybrids
Homoploid bridge
Unilateral pathway
Bilateral pathway (One step)
Gametes
2n
+
2n
2n
+
n
⊗
Somatic chromosome doubling
Neopolyploid (2n = 4x = AABB)
Meiosis
Multivalent formation
Diploidization
Paleopolyploid (2n = 2x = XX)
Meiosis
Bivalent preference
