# Supplementary material for: The evolution of meiotic sex and its alternatives
Source: Proc Biol Sci. 2016 Sep 14;283(1838):20161221. doi: 10.1098/rspb.2016.1221 (PMC5031655; doi:10.1098/rspb.2016.1221)
Supplement: Supplement 4: Mechanisms of sexual and apomictic reproduction in flowering plants. [file rspb20161221supp4.pdf]

Supplement 4: Mechanisms of sexual and apomictic reproduction in flowering plants. The most common form of embryo sac (ES), called the Polygonum type has been depicted in this figure, which is found in 70% of flowering plants. The transition from sporophyte to gametophyte requires the formation of haploid cells (spores) by meiosis, a process called sporogenesis. In flowering plants, sporogenesis starts with the differentiation of sub-epidermal cells in the anther and ovule primordia, called archesporial cells (ACs) into pre-meiotic cells called sporocytes. Both male sporocytes (microsporocytes, also called pollen mother cells or PMCs) and female sporocytes (megasporocytes also called megaspore mother cells or MMCs) will then undergo meiosis to give rise to the microspores and megaspores, in the male and female organs, respectively. Microsporocytes, capable of undergoing meiosis form a tetrad of haploid microspores. Each microspore becomes or male gametophyte followed by two mitoses producing two sperm cells and one vegetative cell within the pollen grain. The sperm nuclei take part in double fertilization of the egg and central cell, leading to the embryo and the endosperm production, respectively. Meiosis in MMC produces four haploid megaspores, only one of which is usually functional (functional megaspore, FM). Followed by three successive mitoses, FM becomes ES which in the Polygonum-type pathway contains two gametes (egg and central cell), two synergids at the micropylar pole and three antipodal cells at the chalazal pole. Double fertilization by two sperms released from the pollen tube results in a diploid zygote and a triploid endosperm cell. In diplospory, restitutional meiosis produces two unreduced spores from MMC, one of which survives and produce ES containing diploid nuclei. While endosperm formation requires fertilization of the 4n central cell in pseudogamous apomicts, the 2n egg develops parthenogenetically into an embryo. Mitotic diplospory also is possible where the

MMC does not enter meiosis. In apospory, aposporous initials (AIs) develop from somatic nucellar tissue nearby the MMC and develop into an unreduced embryo sac. In sporophytic apomixis, a somatic cell from nucellus or integument tissue differentiates directly (not from a gametophyte) into an embryo through a phenomenon called adventitious embryony. In autonomous apomicts, mainly in the family Asteraceae, endosperm formation is pollen-independent. Autonomous apomixis can combine with all three forms of embryo formation.

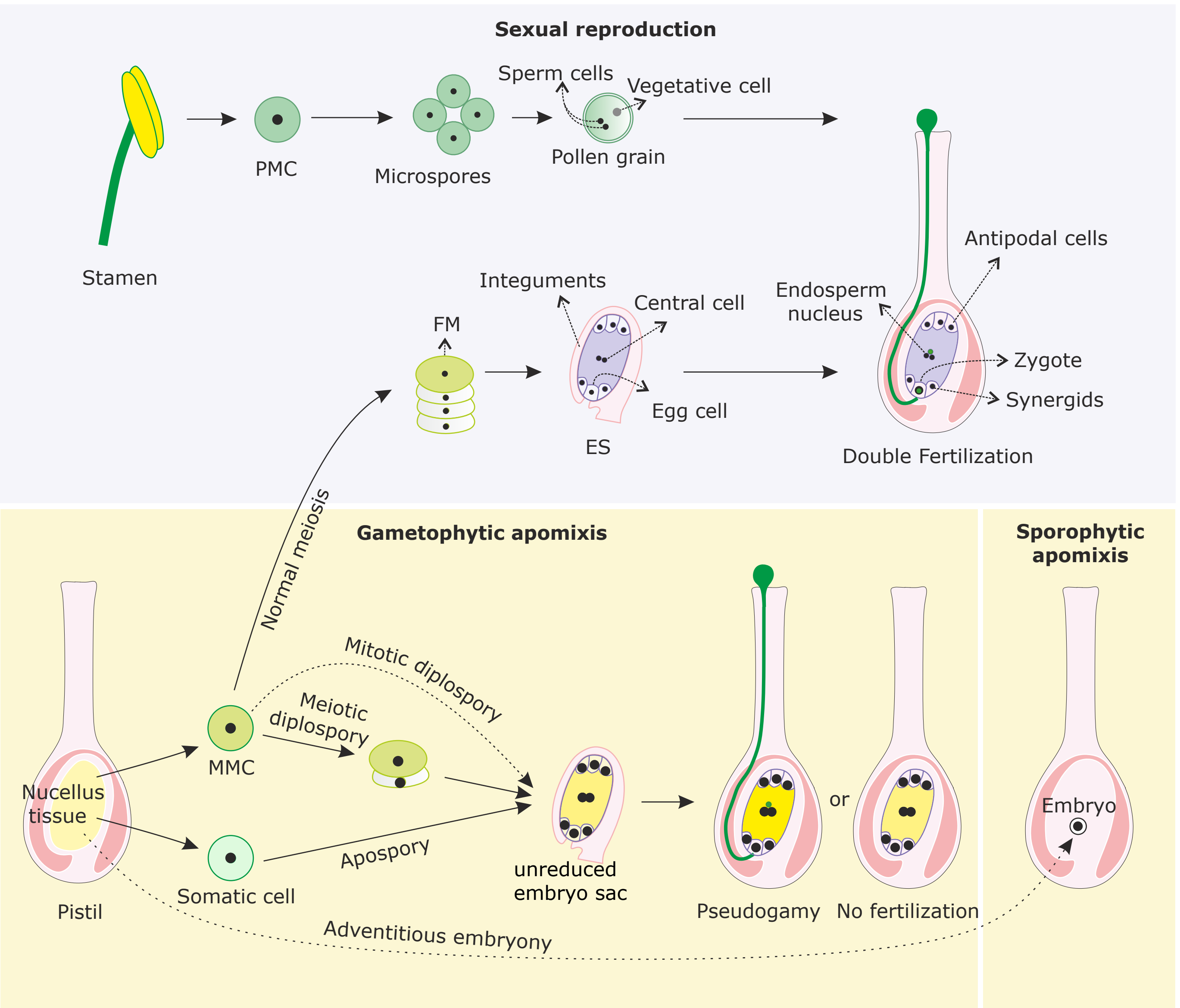
Sexual reproduction
Stamen
PMC
Microspores
Sperm cells
Vegetative cell
Pollen grain
FM
Integuments
Central cell
Egg cell
ES
Endosperm nucleus
Antipodal cells
Zygote
Synergids
Double Fertilization
Gametophytic apomixis
Sporophytic apomixis
Normal meiosis
Mitotic diplospory
Meiotic diplospory
Apospory
MMC
Somatic cell
Nucellus tissue
Pistil
unreduced embryo sac
Pseudogamy
or
No fertilization
Embryo
Adventitious embryony
